# Polyethylene biotransformation by an earthworm gut-derived *Streptomyces* sp.: insights into microenvironment-associated metabolism

**DOI:** 10.1098/rsos.261333

**Published:** 2026-10-14

**Authors:** Morteza Haghi, Tugce Haghi, Torkan Haghi, Zaka Abbaszade

**Affiliations:** ^1^ Department of Research and Development, Bioremedix Biotechnology, Izmir, Turkiye; ^2^ Department of Biology, Atatürk University, Erzurum, Turkiye

**Keywords:** polyethylene degradation, earthworm gut, Fourier transform infrared spectroscopy, genome mining, microenvironment, *Streptomyces* sp

## Abstract

Polyethylene (PE) is a highly recalcitrant polymer that resists microbial degradation owing to its hydrophobic and chemically inert structure. In this study, a *Streptomyces* sp. strain MH-1, isolated from the earthworm gut, was investigated for its interaction with PE under carbon-limited conditions. The strain was associated with approximately 15% reduction in PE weight after 30 days of incubation. Fourier transform infrared (FTIR) analysis revealed alterations in polymer chemistry, including a reduction in the carbonyl peak (approx. 1715 cm^‒1^) and a decrease in the carbonyl index from 0.19 in the control to 0.10 in treated samples. *In silico* analysis using a closely related *Streptomyces* genome as a reference framework identified putative genes associated with hydrocarbon metabolism and suggested potential oxidative and assimilatory pathways. Candidate aldehyde dehydrogenases, including bifunctional alcohol/aldehyde dehydrogenase (*adhE*), together with genes associated with fatty acid metabolism and *β*-oxidation, may indicate potential mechanisms involved in the processing of oxidation intermediates. These findings, combined with biofilm formation and growth under confined conditions, highlight the importance of PE-associated microenvironments and provide a basis for exploring microbial adaptation and potential metabolic processes involved in PE biotransformation.

## 1. Introduction

Plastics are synthetic polymeric materials composed primarily of carbon and hydrogen, often incorporating additional elements such as nitrogen, sulphur and halogens. Owing to their high molecular weight, stable chemical bonds and hydrophobic nature, many plastics exhibit strong resistance to physical, chemical and biological degradation [1,2]. Global plastic production and waste generation have increased dramatically, with annual plastic waste estimated to exceed tens of millions of tons, posing a significant environmental challenge [3].

Among commonly used plastics, polyethylene (PE) is particularly persistent owing to its simple yet highly stable carbon–carbon backbone, which lacks readily hydrolysable functional groups. As a result, its biodegradation under natural conditions is extremely slow [4]. Microbial degradation and transformation of plastics typically involve initial oxidative or enzymatic modification of the polymer surface, followed by fragmentation into smaller molecules such as oligomers and monomers that may be assimilated into microbial metabolic pathways [5,6]. Several enzymes, including oxidases, hydrolases and monooxygenases, have been implicated in these processes [7]. Recent reviews have highlighted major advances in the understanding of microbial, enzymatic and biotechnological mechanisms involved in PE biodegradation and bioconversion, while emphasizing that many metabolic pathways remain incompletely characterized [6].

Microorganisms capable of interacting with or degrading plastics have been isolated from diverse environments, including plastic-contaminated soils, marine systems, landfill sites and the digestive tracts of invertebrates. Recent studies have also reported plastic-associated microbial communities and potential degraders within the earthworm gut, although the underlying metabolic mechanisms remain largely unexplored [8,9]. The earthworm gut represents a confined and dynamic microenvironment characterized by fluctuating oxygen availability, high microbial density and continuous exposure to organic substrates. Such conditions may favour the selection of metabolically versatile microorganisms capable of functioning under diffusion-limited and microaerophilic conditions.

Members of the genus *Streptomyces* are well known for their ability to produce a wide range of extracellular enzymes and to degrade complex organic compounds. However, their role in PE biotransformation has been primarily described at the phenotypic level, and the underlying metabolic mechanisms remain insufficiently explored. In particular, the potential influence of localized microenvironmental conditions, such as oxygen gradients and surface-associated growth, on polymer transformation has received limited attention [10,11].

Despite increasing reports of PE biodegradation, the underlying metabolic pathways remain insufficiently characterized. In this context, *in silico* approaches provide a useful framework to explore the genetic potential of microorganisms for hydrocarbon metabolism. Genome-based analyses enable the identification of enzyme systems potentially involved in the metabolism of polymer-derived intermediates, offering complementary insights to experimental observations. Therefore, combining experimental data with *in silico* gene mining can improve the understanding of microbial interactions with PE. In addition, microenvironmental conditions, such as confined or low-oxygen niches, may influence microbial activity and metabolic responses during polymer biotransformation.

In this study, we isolated bacterial strains from earthworm gut content and evaluated their ability to interact with PE under carbon-limited conditions. A *Streptomyces* isolate showing strong PE colonization and interaction was selected for further analysis. The observed PE colonization and the potential development of localized microenvironmental conditions, including diffusion-limited or microaerophilic niches, provided the basis for exploring possible metabolic adaptations associated with microbial survival and polymer biotransformation. Therefore, experimental observations were integrated with *in silico* genome mining using a closely related near-neighbour *Streptomyces* genome as a hypothesis-generating approach to explore potential metabolic pathways and adaptive strategies associated with PE interaction under localized microenvironmental conditions.

## 2. Material and methods

### 2.1. Earthworm dissection and gut content collection

Adult earthworms were collected from soil and transported to the laboratory in sterile containers. The worms were washed several times with sterile distilled water to remove adhering soil particles. Dissection was performed aseptically inside a biosafety cabinet. Each worm was dissected longitudinally using sterile instruments, and the gut content was aseptically removed. The intestinal material was transferred into sterile tubes containing physiological saline and homogenized for subsequent microbial isolation, following standard procedures [12].

### 2.2. Bacterial isolation and purification

Liquid carbon-free basal medium (LCFBM) was prepared using deionized water and composed of 0.7 g KH_2_PO_4_, 0.7 g K_2_HPO_4_, 0.7 g MgSO_4_·7H_2_O, 1.0 g NH_4_NO_3_, 0.005 g NaCl, 0.002 g FeSO_4_·7H_2_O, 0.002 g ZnSO_4_·7H_2_O and 0.001 g MnSO_4_·H_2_O. The medium was used for the enrichment and isolation of bacteria capable of forming biofilms or degrading plastic materials. For enrichment, 1 g of sterile PE was aseptically added to a 250 ml flask containing 100 ml of LCFBM. Subsequently, 1 ml of gut homogenate was inoculated into the medium and incubated at 25°C for one month. Later, serially diluted samples were inoculated on Luria–Bertani agar medium by the spread plate method and incubated at 25°C for 72 hours. The colonies obtained were purified by the streaking method.

### 2.3. Biofilm formation and screening of isolates

Isolates were inoculated onto LCFBM agar plates containing sterile PE tape using the spread plate method and incubated at 25°C for two weeks. Isolates showing biofilm formation were selected for further PE weight loss and Fourier transform infrared (FTIR) analyses.

### 2.4. Polyethylene weight loss assay

The liquid medium was sterilized by autoclaving at 121°C for 15 minutes. After cooling, 200 ml of the medium was dispensed into sterile 500 ml flasks. Sterile, pre-weighed PE pieces were aseptically transferred into the flasks. Bacterial isolates were adjusted to a turbidity equivalent to 1 McFarland standard and inoculated into the medium. A control flask without bacterial inoculation was maintained under identical conditions. The cultures were incubated at 25°C in a shaker rotating at 120 r.p.m. for 30 days. After incubation, the PE pieces were carefully removed, washed with ethanol followed by distilled water to remove attached biomass, and dried at room temperature. The final weight of the PE was recorded, and the percentage of weight loss was calculated [13–15].

### 2.5. Fourier transform infrared analysis

FTIR spectroscopy analysis was performed to evaluate functional group changes in PE samples. Spectra were recorded in the range of 4000–400 cm^‒1^ for both untreated (control) and bacteria-treated samples. PE samples were obtained after incubation with bacterial isolates under degradation assay conditions. Before analysis, the samples were thoroughly washed with ethanol followed by distilled water to remove attached biomass and residual medium components, and then dried at room temperature [16]. FTIR measurements were carried out at the BIOMER Centre, Izmir Institute of Technology.

### 2.6. Carbonyl index analysis

The extent of oxidative modification of PE was evaluated using the carbonyl index (CI), calculated from FTIR spectra. Absorbance values corresponding to the carbonyl stretching region (approx. 1715 cm^‒1^) and a reference methylene bending peak (1462–1465 cm^‒1^) were obtained from the FTIR spectral data. The CI value was calculated according to the following equation:

$$\begin{array}{r} \text{CI}=A_{1715}/A_{1465}, \end{array}$$

where *A*_1715_ represents the absorbance at approximately 1715 cm^‒1^, and *A*_1465_ represents the absorbance at approximately 1465 cm^‒1^. CI values were calculated for both control and bacteria-treated PE samples and used to assess changes in carbonyl content following microbial treatment [16].

### 2.7. 16s rDNA amplification, sequencing and identification of isolates

On the basis of the preliminary screening and degradation results, a representative isolate exhibiting pronounced biofilm formation and PE transformation capability was selected for molecular identification.

Genomic DNA of the bacterial isolate was extracted by using a commercial kit according to the manufacturer's instructions and visualized by 0.8% agarose gel electrophoresis. The 16S rRNA gene was amplified by polymerase chain reaction (PCR) using universal primers 27F (5′-AGAGTTTGATCCTGGCTCAG-3′) and 1492R (5′-CTACGGCTACCTTGTTACGA-3′). The amplification was performed under the following conditions: initial denaturation at 94°C for 5 minutes, followed by 40 cycles of denaturation at 94°C for 1 minute, annealing at 56°C for 1 minute and extension at 72°C for 1 minute, with a final extension at 72°C for 10 minutes [17].

PCR products were sequenced at the Izmir Institute of Technology, BIOMER Centre. Chromatogram data were quality-trimmed and assembled into a consensus sequence by using Geneious Prime software. The edited sequence was converted to FASTA format and analysed using BLASTn against the National Center for Biotechnology Information (NCBI) 16S rRNA database. The phylogenetic analysis was performed using the neighbour-joining method in MEGA version 12.

### 2.8. *In silico* genome mining and pathway inference

On the basis of the 16S rRNA gene sequence similarity and phylogenetic analysis, a closely related *Streptomyces* near-neighbour genome was selected as a reference framework for *in silico* genome mining. The genome assembly of *Streptomyces albogriseolus* strain NPDC052706 (RefSeq accession: GCF_044901485.1; GenBank accession: GCA_044901485.1; assembly: ASM4490148v1) was retrieved from the NCBI Genome database and used to explore putative genes and potential metabolic pathways associated with PE biotransformation.

The corresponding GBFF file was downloaded and visualized using Geneious Prime software. Genome mining was performed on the basis of functional annotation and keyword-based screening to identify genes potentially associated with PE-associated metabolic processes. Genes related to oxidative processes were investigated, including multicopper oxidases, cytochrome P450 monooxygenases, flavin-containing monooxygenases and alkene reductases.

Enzymes involved in the downstream metabolism of oxidized intermediates were also screened, including alcohol dehydrogenases, aldehyde dehydrogenases and β-oxidation–associated enzymes such as acyl-CoA synthetases and acyl-CoA dehydrogenases.

To evaluate adaptation to microenvironmental conditions, genes associated with oxidative stress response and low-oxygen metabolism were examined, including catalase, superoxide dismutase, peroxiredoxin, glutathione-related enzymes, nitrate reductase and cytochrome bd oxidase components (*cydAB*). In addition, genes encoding lipases and esterases were investigated owing to their potential role in surface interaction with hydrophobic polymer substrates.

The analysis was conducted to infer potential metabolic capabilities rather than to confirm gene presence in the studied isolate.

## 3. Results

### 3.1. Isolation and preliminary screening of polyethylene-associated bacteria

Bacterial strains were isolated from earthworm gut content following enrichment in LCFBM containing PE as the sole carbon source. After incubation and purification, three distinct bacterial isolates were obtained on the basis of colony morphology. Preliminary screening on LCFBM agar plates containing PE tape revealed differences among the isolates in their ability to colonize the polymer.

One isolate, designated MH-1, exhibited pronounced growth with visible biofilm formation and was therefore selected for further analysis (figure 1). Bacterial growth was predominantly observed under the tape and at the edges of the PE surface, suggesting the presence of localized conditions that may influence microbial activity.

**Figure 1.**
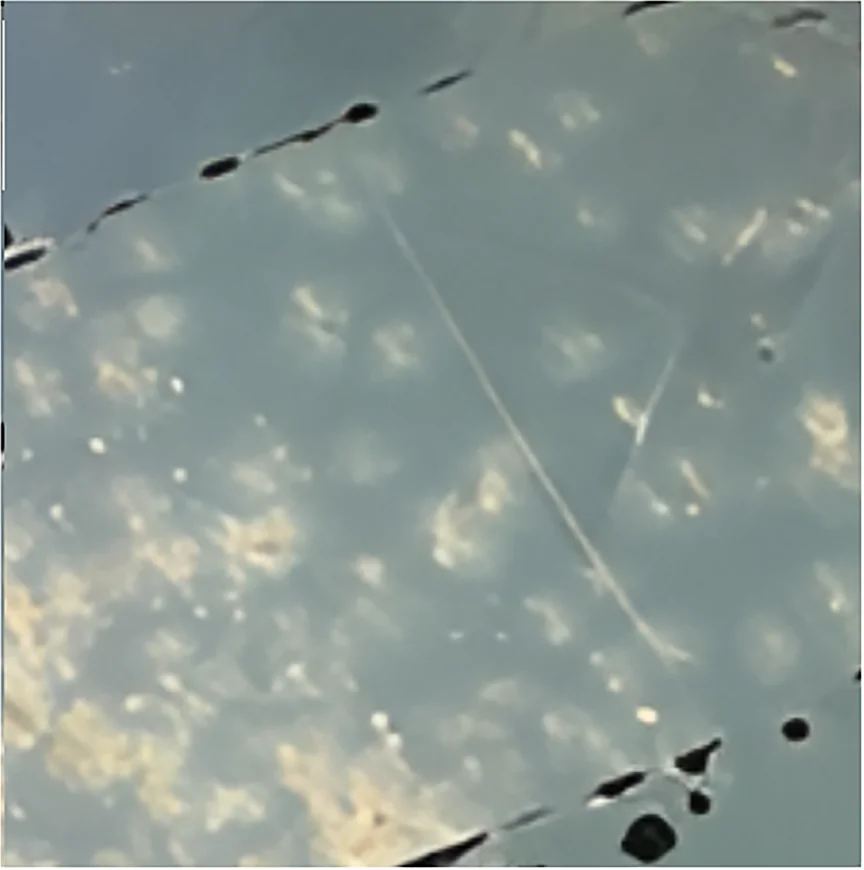
Visible growth pattern of a selected bacterial isolate (MH-1) on PE tape placed on LCFBM agar medium. Bacterial growth was observed predominantly at the edges and beneath the tape on the agar surface, with no visible colonization on the upper surface of the polymer. The image was captured directly from the Petri dish using a smartphone camera without microscopic magnification.

### 3.2. Polyethylene weight loss assay

PE samples inoculated with the isolate MH-1 showed a 15% reduction in weight after the incubation period, whereas no significant change was observed in the uninoculated control.

### 3.3. Molecular identification and phylogenetic analysis

BLASTn analysis showed that isolate MH-1 belonged to the genus *Streptomyces*, with the closest external matches corresponding to *S. albogriseolus* strains at 99.36% sequence identity and 100% query coverage. Accordingly, the isolate was designated as *Streptomyces* sp. MH-1, closely related to *S. albogriseolus*.

Phylogenetic analysis was performed using the neighbour-joining method based on the Tamura–Nei model with 1000 bootstrap replicates. The resulting tree was rooted using *Kitasatospora cinereacea* as the outgroup (figure 2). The multiple sequence alignment used for phylogenetic reconstruction is provided as supplementary material, S1.

**Figure 2.**
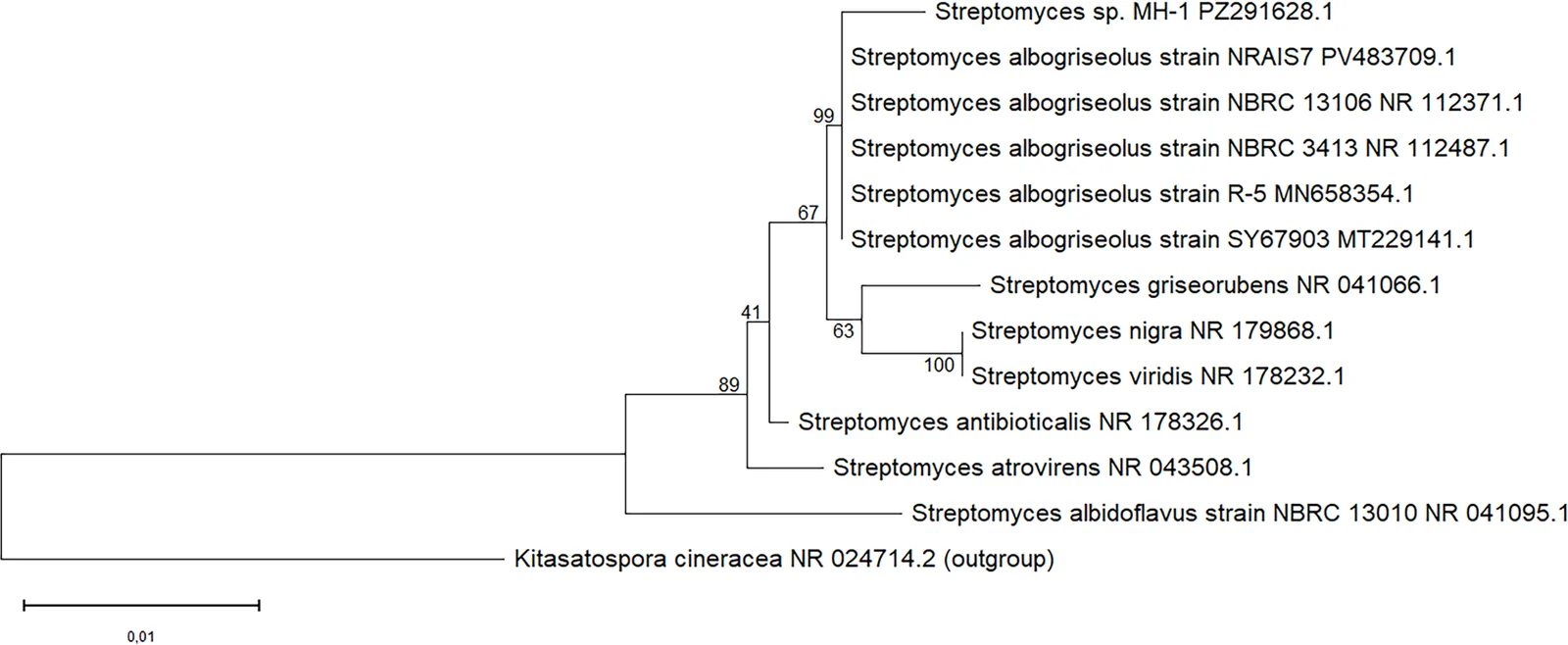
The phylogenetic tree was constructed using the neighbour-joining method. The percentage of replicate trees in which the associated taxa clustered together in the bootstrap test (1000 replicates) is shown next to the branches. The evolutionary distances were computed using the Tamura–Nei method. Evolutionary analyses were conducted in MEGA 12.

### 3.4. Fourier transform infrared and carbonyl index analysis

FTIR spectroscopy was used to evaluate structural changes in PE following treatment with the *Streptomyces* MH-1 strain (figure 3; supplementary material, table S1). Both control and treated samples exhibited characteristic PE absorption bands, including C–H stretching (approx. 2915 and 2848 cm^‒1^) and CH_2_ bending (approx. 1465 cm^‒1^).

**Figure 3.**
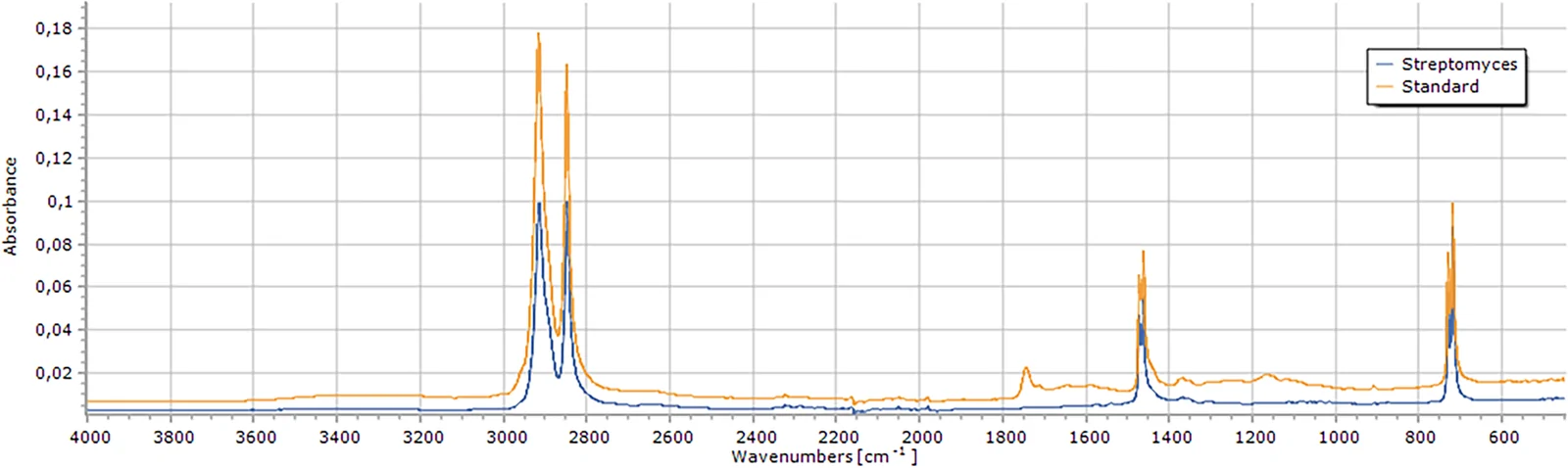
FTIR spectra of PE samples before (control) and after treatment with *Streptomyces* MH-1. Characteristic peaks corresponding to C–H stretching (approx. 2915 and 2848 cm^‒1^), carbonyl stretching (approx. 1715 cm^‒1^) and CH_2_ bending (approx. 1465 cm^‒1^) were observed. A noticeable reduction in the carbonyl peak intensity in the treated sample indicates changes in carbonyl content following microbial treatment.

A distinct difference was observed in the carbonyl stretching region (approx. 1715 cm^‒1^). The control sample showed a higher intensity peak, whereas the *Streptomyces*-treated sample exhibited a noticeable reduction in this band. This decrease indicates a lower relative abundance of carbonyl-containing functional groups following microbial treatment. Minor variations were also observed in the fingerprint region (1000–1300 cm^‒1^), suggesting alterations in oxygen-containing functional groups.

To quantitatively assess these changes, the CI value was calculated as the ratio of absorbance at 1715 cm^‒1^ to that at 1465 cm^‒1^. The CI value for the control sample was determined to be 0.19, whereas the treated sample showed a significantly lower value of 0.1.

### 3.5. *In silico* genome mining and pathway inference

*In silico* analysis revealed the presence of multiple genes associated with hydrocarbon oxidation in the selected reference genome of *S. albogriseolus*. These included genes encoding cytochrome P450 monooxygenases, multicopper oxidases, flavin-containing monooxygenases, as well as enzymes involved in downstream processes such as alcohol dehydrogenases (*adh*), aldehyde dehydrogenases (*aldh*) and genes associated with β-oxidation pathways. In addition, genes related to fatty acid metabolism and β-oxidation pathways were identified (supplementary material, table S2).

## 4. Discussion

PE biodegradation is considered a more environmentally sustainable alternative to conventional disposal methods such as incineration and landfilling, which contribute to environmental pollution and disruption of natural cycles [18]. However, the intrinsic properties of PE, including its high molecular weight, hydrophobicity and lack of functional groups, make it resistant to microbial degradation [5].

Gut-associated microbial communities may also represent a reservoir of PE-transforming micro organisms. Bacteria isolated from earthworm gut environments have been reported to contribute to low-density PE (LDPE) degradation, whereas similar capabilities have been observed in gut-derived systems such as insect larvae [19,20].

In the present study, *Streptomyces* sp. MH-1 exhibited the capacity to interact with and biotransform PE, resulting in a 15% reduction in polymer weight after 30 days of incubation. This value is comparable to previously reported degradation rates under similar conditions. For example, Harshvardhan & Jha [21] reported weight losses of 1%–1.75% after 30 days, whereas Balasubramanian *et al.* [22] observed degradation levels of 12%–15% using *Arthrobacter* and *Pseudomonas*. Similar findings have also been reported for *Streptomyces* spp. and *Klebsiella pneumoniae* [10,23,24].

Although weight loss measurements alone do not establish complete PE biodegradation or mineralization, the combined observations of polymer weight reduction, FTIR alterations and biofilm formation may suggest microbial interaction with PE and structural modification of the polymer under the experimental conditions used.

FTIR analysis revealed alterations in the chemical structure of PE following treatment with *Streptomyces* sp. MH-1. In particular, a reduction in the intensity of the carbonyl peak (approx. 1715 cm^‒1^) was observed, accompanied by a decrease in the CI value from 0.19 in the control to 0.10 in the treated sample. This decrease in carbonyl content suggests that oxidized intermediates formed during PE transformation may have been further metabolized rather than accumulated. Supporting this observation, reference genome-based analysis identified multiple genes encoding enzymes involved in hydrocarbon metabolism, including cytochrome P450 monooxygenases, alcohol dehydrogenases and aldehyde dehydrogenases. These enzymes are known to function sequentially in the oxidation of hydrocarbon substrates, converting initial oxidation products into alcohols, aldehydes and subsequently into corresponding acids [25].

The identified putative enzymatic systems in the near-neighbour genome may indicate metabolic pathways involved in the further processing of PE-derived intermediates. Aldehyde dehydrogenases, including bifunctional alcohol/aldehyde dehydrogenase (*adh*E), may contribute to the conversion of carbonyl-containing compounds into downstream metabolites, potentially explaining the observed reduction in carbonyl signal detected by FTIR. In addition, genes associated with fatty acid metabolism and *β*-oxidation suggest that transformed intermediates could be used as carbon and energy sources. Together, the FTIR observations and reference genome-based analysis provide preliminary mechanistic insights into possible metabolic processes associated with PE transformation.

Although this approach does not confirm the gene content of *Streptomyces* sp. MH-1, it identifies candidate pathways for future experimental validation. Similar near-neighbour genome-based approaches have previously been used to infer functional capabilities and generate hypotheses regarding the metabolic potential and ecological adaptations of unsequenced environmental microorganisms [26–28].

The observed colonization of the PE and biofilm formation may indicate localized conditions that influence microbial activity. Such microenvironments could potentially affect oxygen availability and microbial activity. These localized conditions may not only influence surface colonisation but also modulate metabolic activity, particularly oxygen-dependent oxidation processes involved in PE transformation. Variations in oxygen availability at the polymer interface could influence the balance between initial oxidation and subsequent metabolic conversion steps, potentially contributing to the observed changes in carbonyl content. This behaviour may be consistent with the environmental conditions experienced by the isolate, as the earthworm gut represents a transient microenvironment derived from soil, characterized by heterogeneous and microaerophilic conditions that may influence microbial activity. PE transformation by *Streptomyces* species has been previously reported; studies integrating experimental observations with *in silico* approaches to explore microenvironment-associated metabolic adaptation remain limited. The findings of the present study contribute to an understanding of how localized environmental conditions may influence microbial survival, adaptation and PE biotransformation.

## 5. Conclusion

This study demonstrates that a *Streptomyces* sp. MH-1 isolated from earthworm gut content can interact with PE under carbon-limited conditions and contribute to its biotransformation. The objective of the present study was not to establish complete PE biodegradation, but rather to investigate microbial interactions with PE and explore possible metabolic adaptations associated with localized microenvironmental conditions. The integration of experimental observations and *in silico* analyses provides a hypothesis-generating framework for understanding potential mechanisms involved in microbial adaptation and polymer-associated metabolic processes. FTIR analysis revealed a reduction in carbonyl peak intensity and CI, indicating modification of the PE material and possible biotransformation of oxidized intermediates. Analysis using a closely related near-neighbour genome identified candidate enzymes associated with hydrocarbon metabolism and provided insights into potential oxidative and assimilatory pathways.

Overall, these findings contribute to a better understanding of microbial–polymer interactions and support future investigations into microenvironment-associated mechanisms and gut-inspired approaches for plastic biotransformation.

## Supplementary Material



## Data Availability

The 16S rRNA gene sequence of the *Streptomyces* sp. isolate has been deposited in GenBank under accession number PZ291628.1. Supporting data associated with this study are provided as supplementary material [29].
